# The Reaction Mechanism of Claisen Rearrangement Obtained by Transition State Spectroscopy and Single Direct-Dynamics Trajectory

**DOI:** 10.3390/molecules18021995

**Published:** 2013-02-04

**Authors:** Izumi Iwakura, Yu Kaneko, Shigehiko Hayashi, Atsushi Yabushita, Takayoshi Kobayashi

**Affiliations:** 1 Innovative Use of Light and Materials/Life, PREST, JST, 4-1-8 Honcho, Kawaguchi, Saitama 332-0012, Japan; 2 University of Electro-Communications, 1-5-1 Chofugaoka, Chofu, Tokyo 182-8585, Japan; 3 Department of Chemistry, Graduate School of Science, Kyoto University, Kitashirakawa, Sakyo, Kyoto 606-8502, Japan; E-Mails: yukan280@gmail.com (Y.K.); hayashig@kuchem.kyoto-u.ac.jp (S.H.); 4 Department of Electrophysics, National Chiao-Tung University, Hsinchu 300, Taiwan; E-Mail: yabushita@mail.nctu.edu.tw (A.Y.)

**Keywords:** Claisen rearrangement, 5-fs pulse laser, reaction mechanism

## Abstract

Chemical bond breaking and formation during chemical reactions can be observed using “transition state spectroscopy”. Comparing the measurement result of the transition state spectroscopy with the simulation result of single direct-dynamics trajectory, we have elucidated the reaction dynamics of Claisen rearrangement of allyl vinyl ether. Observed the reaction of the neat sample liquid, we have estimated the time constants of transformation from straight-chain structure to aromatic-like six-membered ring structure forming the C^1^-C^6^ bond. The result clarifies that the reaction proceeds via three steps taking longer time than expected from the gas phase calculation. This finding provides new hypothesis and discussions, helping the development of the field of reaction mechanism analysis.

## 1. Introduction

Phenomena which are too fast to be directly observed by our eye, can be visualized by observing them using strobe lights. The use of such stroboscopic method to observe ultrafast chemical bond breaking and formation in chemical reactions has been a long-awaited dream for chemists. Lord G. Porter was awarded Nobel Prize in Chemistry for his contribution to the technique of flash photolysis [1].

After the first report of laser oscillation in 1960 [2], laser pulses have been used as strobe lights and its time duration has been kept trying to be shortened as short as attosecond order [3,4,5]. In the field of ultrafast optical measurement, the shorted pulse of femtosecond strobe light enabled us to observe electronic and vibration spectra in transition states of photoreactions. Zewail, who was awarded the Nobel Prize in Chemistry for his pioneering work on femtosecond time-resolved spectroscopy [6], has proposed “transition state spectroscopy” as a study of transition state realizing the chemists dream to observe chemical bond breaking and formation. Generally, heavy atom—hydrogen stretching vibrational modes (3000–3800 cm^−1^) have a period of 11–9 fs, and carbonyl stretching vibrational mode and C=C bond stretching vibrational mode (1600–1750 cm^−1^) show the vibration in a period of 21–19 fs. Therefore, using laser pulses whose duration is much shorter than the vibration periods, molecular motion in those vibrational modes can be time-resolved observing the modulation of transition probability of the corresponding wavelength in real-time. It means that molecular structure changes in photoreactions can be observed by measuring the real-time amplitudes of molecular vibrations from which time-dependent frequencies are calculated [7,8,9]. In addition, we have previously reported [8,9,10,11] that when the pump photon energy is lower than the minimum electronic transition energy, molecular vibrational modes of the electronic ground state are excited via an induced Raman process, which triggers the reaction in the electronic ground state without converting photon energy to thermal energy. As a result, thermally allowed reactions also can be observed by measuring the real-time amplitudes of molecular vibrations, from which time-dependent frequencies are calculated. In this work, a visible 5-fs laser pulse, which is much shorter than those vibration periods, was used to observe molecular structure changes in thermally allowed Claisen rearrangements, including their transition states.

## 2. Results and Discussions

### 2.1. Transition State Spectroscopy of the Claisen Rearrangement of Allyl Vinyl Ether

**Figure 1:**
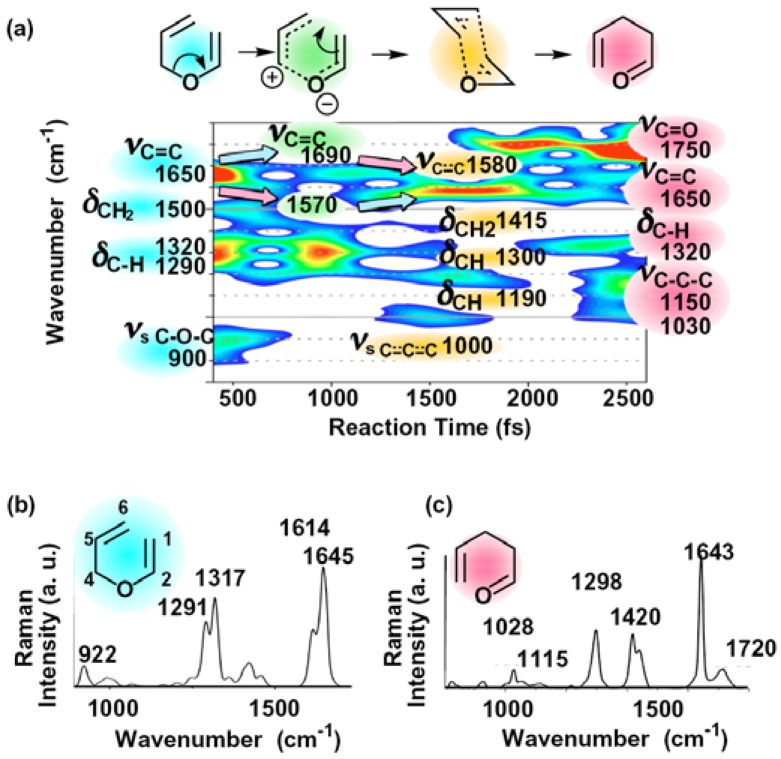
(**a**) Spectrogram of a Claisen rearrangement induced by visible 5 fs pulses [10]; (**b**) Raman spectrum of AVE; (**c**) Raman spectrum of allyl acetaldehyde.

We have performed pump-probe measurement of neat liquid of allyl vinyl ether (AVE). Measured time-resolved absorption change traces were analyzed by time-frequency analysis [12] using a Blackmann window function of 400 fs FWHM. The result is shown in Figure 1a [10], whose x-axis and y-axis correspond to reaction time after 5 fs pulse irradiation and time dependent molecular vibration frequency, respectively. In the spectrogram, the molecular vibrational modes appear immediately after the 5 fs pulses irradiation being assigned to those of AVE; C-O-C symmetric stretching vibrational mode (*ν*_s C–O–C_) of the ether group (900 cm^−1^), C-H deformation vibrational modes (*δ*_C–H_) of the allyl and the vinyl groups (1290 cm^−1^ and 1320 cm^−1^, respectively), C–H_2_ deformation vibrational mode (*δ*_C–H__2_) of the methylene group (1500 cm^−1^) and C=C stretching vibrational modes (*ν*_C=C_) of the allyl and the vinyl groups (1650 cm^−1^). These molecular vibrational modes agree well with the Raman data of AVE (Figure 1b).

The CH_2_ deformation vibrational mode of the methylene group and the C–O–C symmetric stretching vibrational mode of the ether group disappear at about 800 fs delay. It implies that the C^4^-O bond is weakened or broken in the first step of the reaction. The wavenumber shifts of the C=C bonds stretching modes also suggest that the C^4^-O bond is weakened. Just after the 5 fs pulses irradiation, the C=C bond stretching vibrational mode of the vinyl and that of the allyl groups appear around 1650 cm^−1^. Then, the C^4^-O bond weakening causes the electronic density of the allyl and the vinyl groups to decrease and increase, respectively. Therefore, the C=C bond stretching vibrational mode observed at 1650 cm^−1^ was separated into a red-shifted mode toward 1570 cm^−1^ and in a blue-shifted mode toward 1690 cm^−1^.

After the C^4^-O bond weakening, electrons transfer from the vinyl group to the allyl group to form a weak C^1^-C^6^ bond, which causes an increase and decrease of the electronic density of the allyl and the vinyl groups, respectively. Thus, the C^5^=C^6^ bonds stretching vibrational mode of the allyl group is blue-shifted from 1570 to 1580 cm^−1^, and the C^1^=C^2^ bonds stretching vibrational mode of the vinyl group is red-shifted from 1690 to 1580 cm^−1^. In addition, the electron transfer from the vinyl group to the allyl group makes the C=C bonds of both of the allyl and the vinyl groups equivalent having the same wavenumber of 1580 cm^−1^ around 1500 fs delay, which implies that aromatic-like C=C bonds are formed. This result shows that the generated intermediate has an aromatic-like six-membered structure. Finally, C^4^-O bond breaking and C^1^-C^6^ bond formation proceed simultaneously to generate allyl acetaldehyde being observed in appearance of molecular vibrational modes around 2,000 fs delay. The frequencies of the new modes at 1030, 1150 and 1750 cm^−1^ agree well with the Raman data of allyl acetaldehyde (Figure 1c), which can be assigned to the C-C-C symmetric stretching vibrational mode (*ν*_s C–C–C_), the C–C–C asymmetric stretching vibrational mode (*ν*_as C–C–C_), and the C=O stretching vibrational mode (*ν*_C=O_), respectively.

**Figure 2:**
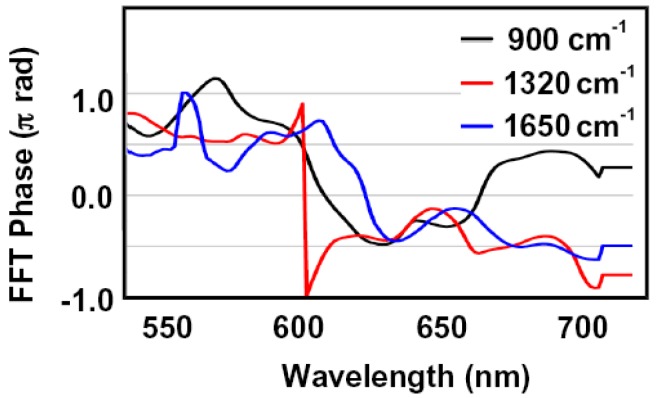
Fourier initial phase spectra of 900 cm^−1^ (black line), 1320 cm^−1^ (red line), and 1650 cm^−1^ (blue line) modes.

In addition, the initial phases of the observed molecular vibrational modes appeared immediately after the 5 fs pulses irradiation (900, 1320, and 1650 cm^−1^) are close to sine-like within ±0.21 radian (Figure 2). Therefore, this result conforms that the observed molecular vibrational modes are associated with the wavepacket in the ground state.

**Figure 3:**
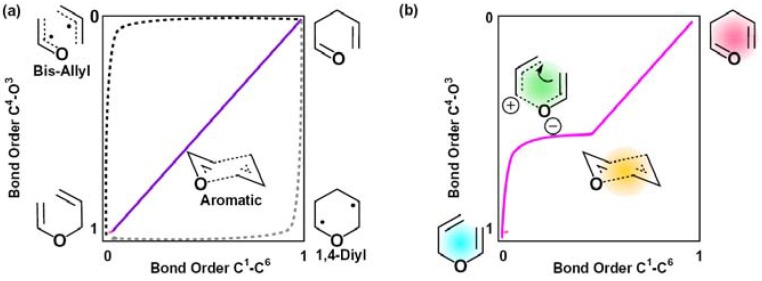
Transition-state profile for the Claisen rearrangement. (**a**) Purple curve: a synchronous concerted pathway reaction *via* an aromatic-like TS. Black curve: a stepwise pathway reaction *via* a bis-allyl like TS. Gray curve: a stepwise pathway reaction *via* a 1-4-diyl like TS; (**b**) Red curve: this work.

[3,3]-Sigmatropic rearrangements of allyl aryl ethers were reported by Claisen in 1912 [13], which has been followed by broad variations of the Claisen rearrangements as below. After the first report of [3,3]-sigmatropic rearrangements of allyl vinyl ether in 1938 by Schuler and Murphy [14] and its first kinetic study [15], the [3,3]-sigmatropic rearrangement of allyl vinyl ether has been widely studied as a most simple model of the Claisen rearrangement in kinetic isotope effect measurements in experiments and reaction mechanism analysis in theoretical calculations [16,17,18,19,20,21,22,23,24,25,26,27,28,29,30,31]. In the transition state of the Claisen rearrangement, substitution and solvent were reported to affect the competing processes of C^4^-O bond breaking and C^1^-C^6^ bond formation, resulting in changes of the detailed structure of the transition states [32]. In general, three possible mechanisms have been suggested for the Claisen rearrangement mechanism of allyl vinyl ether [16,17,18,19,20,21,22,23,24,25,26,27,28,29,30,31]. In the first mechanism, the reaction proceeds in a synchronous concerted pathway *via* an aromatic-like transition state. The second possible mechanism proposes an asynchronous stepwise pathway *via* a bis-allyl like transition state, in which C^4^-O bond breaking takes place in the first step of the reaction. The third possible mechanism indicates an asynchronous stepwise pathway *via* a 1-4-diyl-like transition state, in which C^1^-C^6^ bond formation takes place in the first step of the reaction. In this work, we have observed intermediates and transition states which indicate a new possible mechanism for the Claisen rearrangement. The mechanism is described by a three-step pathway. At first, the C^4^-O bond is weakened to generate a bis-allyl-like intermediate. Next, the formation of a weak C^1^-C^6^ bond results in the generation of an aromatic-like six-membered intermediate. Finally, C^4^-O bond breaking and C^1^-C^6^ bond formation occur simultaneously to generate allyl acetaldehyde (Figure 3). This resembles the transiton state reported in the Claisen rearrangement of alkoxy allyl enol ether [33].

In any general thermally activated reaction, the reactant spends a substantial part of the total reaction time waiting for the very rare circumstance to get sufficient energy in the reaction coordinate. Once it does, the rest of the reaction is very fast. In other words, the reaction does not progress along the reaction coordinate at constant speed. However, the calculated spectrogram in Figure 1 showed that the reaction progressed in a different way as follows. The first step of the reaction (generation of a bis-allyl-like intermediate) proceeds in 800–1,000 fs. The second step generates an aromatic-like six-membered intermediate in 300–500 fs. The final step of the three-step pathway finishes in several tens to several hundreds femtoseconds. The observed reaction timescale was confirmed by theoretical calculation of single direct-dynamics trajectory.

### 2.2. Single Direct-Dynamics Trajectory

**Figure 4:**
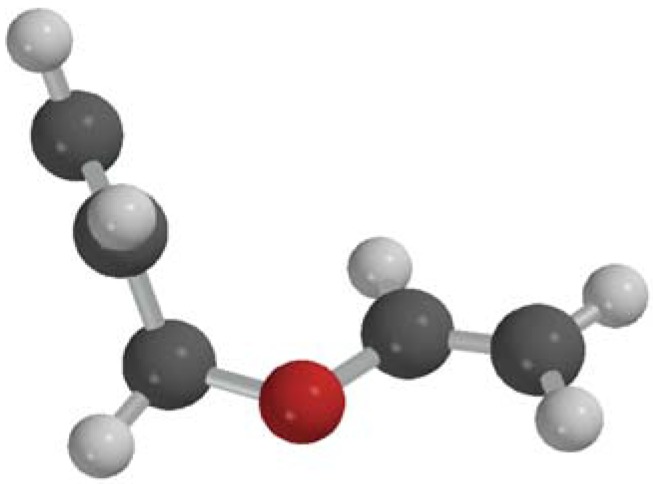
Bow-like structured AVE obtained in the IRC calculation.

Molecular dynamics of the Claisen rearrangement of AVE was simulated by dynamic reaction coordinate (DRC) calculations with large kinetic energy equally assigned to all degrees of freedom of the molecule in an isolated condition. The calculations were performed with GAMESS 2009 program package [34]. Trajectories were computed at the B3LYP/6-311G+(d,p) level of theory. Excess kinetic energy of 8–12 kcal/mol was provided for each freedom degree in the calculation. Several trajectory calculations with different directions of initial velocities generated randomly were carried out. A bow-like structured AVE obtained in the intrinsic reaction coordinate (IRC) calculation was used as an initial structure of the DRC calculation (Figure 4).

**Figure 5:**
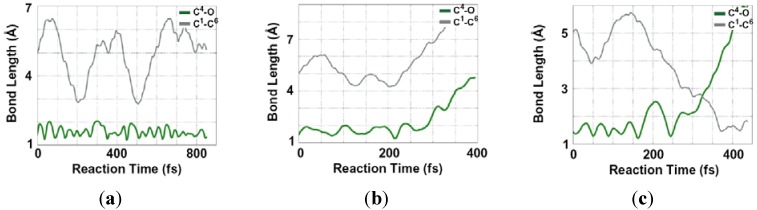
Bond length changes of C^4^-O bond (green curve) and C^1^-C^6^ bond (gray curve) observed in the DRC trajectories with initial kinetic energies of (**a**) 8 kcal/mol, (**b**) 10 kcal/mol, and (**c**) 12 kcal/mol.

Figure 5 summarizes typical time evolutions of length of bonds that dissociates (C^4^-O) and forms (C^1^-C^6^) in the Claisen rearrangement. A successful trajectory which underwent the Claisen rearrangement was observed in a calculation with the initial kinetic energy of 10 kcal/mol (Figure 5b). Dissociation of C^4^-O bond and decrease of distance between C^1^ and C^6^ lead to the aromatic-like six membered structure around 320 fs after large elongation and recovery of the dissociating bond length at 200 fs.

**Figure 6:**
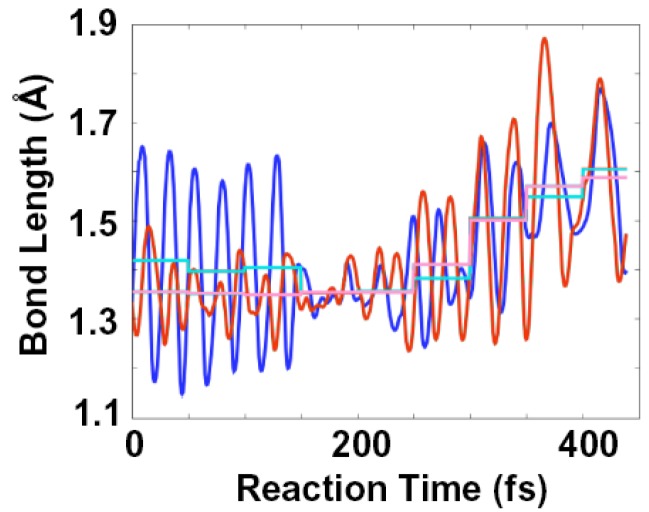
Time evolutions of bond lengths of C^1^=C^2^ (blue curve) and C^5^=C^6^ (red curve) in the trajectory with initial kinetic energy of 10 kcal/mol. Means in 50 fs periods are also indicated (light blue and pink lines, respectively).

Time evolutions of bond lengths of C^1^=C^2^ and C^5^=C^6^ in the reactive trajectory (Figure 6) indicate that the first structure of the trajectory calculation shown in Figure 4 corresponds to the structure in the 800–1,000 fs time region of the spectrogram. Those bond lengths are observed to be distinctly different, ~1.35 and ~1.42Å, respectively, during 0 fs to 200 fs, which agree with C=C stretching modes appearing at 1690 and 1570 cm^−1^ around 1,000 fs after photo-excitation in the spectrogram.

After the initial phase, those bond lengths in the trajectory become almost identical when C^4^-O bond starts to break and the aromatic-like six-membered structure forms during 200 fs to 320 fs. This behavior is consistent with merge of the two C=C stretching bands to a single band of the aromatic-like six-membered structure appearing at 1580 cm^−1^ around 1,500 fs delay in the spectrogram.

In the reactive trajectory, the product is generated within ~150 fs after the formation of the aromatic-like six-membered structure. The time constant is half of that observed in the spectrogram. The discrepancy arises presumably due to the isolated condition of the trajectory calculation which lacks interaction with surrounding molecules. Molecular friction caused by the interaction with surrounding molecules in neat solvent used in the experiment could slow the product formation. 

In the case of the initial kinetic energy of 8 kcal/mol, the C^4^-O bond did not dissociate and in turn the Claisen rearrangement was not observed (Figure 5a). Although C^1^ and C^6^ atoms approached closely during the trajectory, thermal elongation of C^4^–O bond was not enough to initiate the rearrangement. On the other hand, when the initial kinetic energy of 12 kcal/mol was supplied, the larger excess energy led to fragmentation, *i.e.*, C^4^-O bond dissociation without bond formation between C^1^ and C^6^ atoms (Figure 5c). However, the fragmentation would be strongly suppressed in neat solvent due to caging effect. Furthermore, an NMR measurement confirmed that the product was pure allyl acetaldehyde, whereas such fragmentations are expected to generate various product species. We therefore conclude that the rearrangement proceeds through the aromatic-like six membered structure as observed in the trajectory with the initial kinetic energy of 10 kcal/mol.

## 3. Experimental

### 3.1. Visible 5-fs Laser System

The ultrashort pulse laser [35] and ultrafast spectroscopy system used in the measurement is described elsewhere [36] and it is briefly summarized in the following. The output pulse from a Ti:sapphire regenerative amplifier (Spectra Physics Spitfire) with 100 fs duration, centered at 790 nm, and 5 kHz repetition rate was separated by a beam splitter in two pulses. One of the two pulses was focused into a β-BaB_2_O_4_ (BBO) crystal (0.4 mm-thick, θ = 29°) to generate second harmonic (SH) pulse, which was used as a pump pulse in the following optical parametric amplification. The other pulse of the separated two pulses was focused in a 2mm-thick sapphire plate to generate femtosecond white light broadening spectral bandwidth by third order nonlinear effect of self phase modulation. The white light pulse was amplified in a non-collinear optical parametric amplifier (NOPA) pumped by the SH pulse. In the NOPA, the angle between the SH pump pulse and the white light seed pulse was set to be 3.7 degrees in the non-linear crystal (type-I BBO crystal, 1 mm-thick, θ = 31.5°) to satisfy the phase matching condition in broad visible spectral region, which results in broadband amplification of the white light seed pulse. A prism pair and a chirped mirror pair were used to compensate material dispersion compressing the pulse duration as short as 5 fs to be used for pump-probe measurement. The pulse duration can be compressed more as short as 3.9 fs inserting an additional chirp compressor made of a diffraction grating and a deformable mirror.

### 3.2. “The Reaction in the Electronic Ground State”, Triggered by the Visible 5-fs Pulse

Most organic compounds have absorption bands in the ultraviolet region, therefore the visible 5 fs pulse with broad bandwidth of 525–725 nm does not excite their electronic states by single photon excitation, but induces their molecular vibration via stimulated Raman process with Λ-type interaction in the ground state or V-type interaction in the excited state [37]. The 5 fs pulse has a bandwidth of 5200 cm^−1^, which can excite high energy vibration bands as high as in the case of thermal excitation at 7,500 K. Meanwhile, being different from the standard thermal reaction, certain vibration modes are selectively excited by selection rule and cross section of the stimulated Raman process. Activated vibration modes have high vibration quantum number comparable with that of thermal excitation at 7,500 K, while other vibration modes keep the low vibration quantum number of that at room temperature. This direct excitation of vibration modes and its relaxation and transfer to other modes in several hundred femtoseconds [38] will excite vibration modes on the reaction pathway. Thus, this impulsive excitation by the visible 5 fs pulse triggers “the reaction in the electronic ground state”, which is different from that induced by electronic state excitation under photo irradiation conditions.

## 4. Conclusions

In conclusion, the Claisen rearrangement of allyl vinyl ether was triggered by a new scheme exciting the sample by visible 5 fs pulses whose photon energy is much lower than the absorption band of the sample. Observing the molecular vibration frequency changes in the reaction, including its transition states, elucidated the reaction mechanism of the Claisen rearrangement in the new scheme. The time constants of transformation from straight-chain structure to aromatic-like six-membered ring structure forming the C^1^-C^6^ bond were estimated from the observed dynamics of the molecular vibration modes. It was compared with the molecular dynamics simulated by dynamic reaction coordinate calculations with large kinetic energy. The result clarifies that the reaction proceeds via three steps showing agreement between the observed molecular vibration frequency change and that predicted in the dynamic reaction coordinate calculations. This finding provides a new hypothesis and discussions, helping the development of the field of reaction mechanism analysis.

## References

[1] Norish R.G.W., Porter G. (1949). Chemical reactions produced by very high light intensities. Nature.

[2] Mayman T.H. (1960). Stimulated optical radiation in ruby. Nature.

[3] Hentschel M., Kienberger R., Spielmann Ch., Reider G.A., Milosevic N., Brabec T., Corkum P., Heinzmann U., Drescher M., Krausz F. (2001). Attosecond metrology. Nature.

[4] Itatani J., Levesque J., Zeidler D., Niikura H., Pépin H., Kieffer J.C., Corkum P.B., Villeneuve D.M. (2004). Tomographic imaging of molecular orbitals. Nature.

[5] Krausz F., Ivanov M. (2009). Attosecond physics. Rev. Mod. Phys..

[6] Zewail A.H. (2000). Femtochemistry: Atomic-Scale Dynamics of the Chemical Bond. J. Phys. Chem. A.

[7] Kobayashi T., Saito T., Ohtani H. (2001). Real-time spectroscopy transition state in bacteriodhodopsin during retinal isomerization. Nature.

[8] Iwakura I. (2011). The experimental visualization of molecular structural changes during both photochemical and thermal reactions by real-time vibrational spectroscopy. Phys. Chem. Chem. Phys..

[9] Kobayashi T., Yabushita A. (2011). Transition-state spectroscopy using ultrashort laser pulses. Chem. Rec..

[10] Iwakura I., Yabushita A., Kobayashi T. (2010). Direct observation of the molecular structural changes during the Claisen Rearrangement including the transition state. Chem. Lett..

[11] Iwakura I., Yabushita A., Liu J., Okamura K., Kobayashi T. (2012). Photo-impulsive reactions in the electronic ground state without electronic excitation: Non-photo, Non-thermal chemical reactions. Phys. Chem. Chem. Phys..

[12] Vrakking M.J.J., Villeneuve D.M., Stolow A. (1996). Observation of fractional revivals in molecular wavepackets. Phys. Rev. A.

[13] Claisen L. (1912). Rearrangement of phenol allyl ethers into C-allylphenols. Chem. Ber..

[14] Hurd C.D., Pollack M.A. (1938). The rearrangement of vinyl allyl ethers. J. Am. Chem. Soc..

[15] Schuler F.W., Murphy G.W. (1950). The kinetics of the rearrangement of vinyl allyl ether. J. Am. Chem. Soc..

[16] Castro A.M.M. (2004). Claisen rearrangement over the past nine decades. Chem. Rev..

[17] Gajewski J.J., Jurayj J., Kimbrough D.R., Gande M.E., Ganem B., Carpenter B.K. (1987). The mechanism of rearrangement of chorismic acid and related compounds. J. Am. Chem. Soc..

[18] Vance R.L., Rondan N.G., Houk K.N., Jensen F., Borden W.T., Komornicki A., Wimmer E. (1988). Transition structures for the Claisen rearrangement. J. Am. Chem. Soc..

[19] Wiest O., Black K.A., Houk. K.N. (1994). Density functional theory isotope effects and activation energies for the cope and Claisen rearrangements. J. Am. Chem. Soc..

[20] Hu H., Kobrak M.N., Xu C., Hammes-Schiffer S. (2000). Reaction path Hamiltonian analysis of dynamical solvent effects for a Claisen rearrangement and a Diels Alder reaction. J. Phys. Chem. A.

[21] Hill J.G., Karadakov P.B., Cooper D.L. (2006). A spin-coupled study of the Claisen rearrangement of allyl vinyl ether. Theor. Chem. Acc..

[22] Dewar M.J.S., Healy E.F. (1984). Ground states of molecules. 68. MNDO study of the Claisen rearrangement. J. Am. Chem. Soc..

[23] Dewar M.J.S., Jie C. (1989). Mechanism of the Claisen rearrangement of allyl vinyl ethers. J. Am. Chem. Soc..

[24] Gajewski J.J., Conrad N.D. (1979). Aliphatic Claisen rearrangement transition state structure from secondary .alpha.-deuterium isotope effects. J. Am. Chem. Soc..

[25] Gajewski J.J., Gee K.R., Jurayj J. (1990). Energetic and rate effects of the trifluoromethyl group at C-2 and C-4 on the aliphatic Claisen rearrangement. J. Org. Chem..

[26] Kupczyk-Subotkowska L., Saunders W.H., Shine H.J., Subotkowski W. (1994). Carbon kinetic isotope effects and transition structures in the rearrangements of allyl vinyl ethers. 2-(Trimethylsilyloxy)- and 2-(Methoxycarbonyl)-3-oxa-1,5-hexadiene. J. Am. Chem. Soc..

[27] Davidson M.M., Hillier I.H. (1995). Aqueous acceleration of the Claisen rearrangement of allyl vinyl ether: A hybrid, explicit solvent, and continuum model. J. Phys. Chem..

[28] Gajewski J.J. (1997). The Claisen rearrangement. Response to solvents and substituents: The case for both hydrophobic and hydrogen bond acceleration in water and for a variable transition state. Acc. Chem. Res..

[29] Aviyente V., Yoo H.Y., Houk K.N. (1997). Analysis of substituent effects on the Claisen rearrangement with Ab Initio and density functional theory. J. Org. Chem..

[30] Yoo H.Y., Houk K.N. (1997). Theory of substituent effects on pericyclic reaction rates: Alkoxy substituents in the Claisen rearrangement. J. Am. Chem. Soc..

[31] Meyer M.P., DelMonte A.J., Singleton D.A. (1999). Reinvestigation of the isotope effects for the Claisen and aromatic Claisen rearrangements: The nature of the Claisen transition states. J. Am. Chem. Soc..

[32] Rehbein J., Hiersemann M., Hiersemann M., Nubbemeyer U. (2007). Mechanistic Aspects of the Aliphatic Claisen Rearangement. The Claisen Rearrangement.

[33] Coates R.M., Rogers B.D., Hobbs S.J., Curran D.P., Peck D.R. (1987). Synthesis and Claisen rearrangement of alkoxyallyl enolethers. Evidence for a dipolar transition state. J. Am. Chem. Soc..

[34] Schmidt M.W., Baldridge K.K., Boatz J.A., Elbert S.T., Gordon M.S., Jensen J.H., Koseki S., Matsunaga N., Nguyen K.A., Su S.J. (1993). General atomic and molecular electronic structure system. J. Comput. Chem..

[35]  Shirakawa A., Sakane I., Takasaka M., Kobayashi T. (1999). Sub-5-fs visible pulse generation by pulse-front-matched noncollinear optical parametric amplifier. Appl. Phys. Lett..

[36] Kobayashi T., Iwakura I., Yabushita A. (2008). Excitonic and vibrational nonlinear processes in a polydiacetylene studied by a few-cycle pulse laser. New J. Phys..

[37] Du J., Teramoto T., Nakata K., Tokunaga E., Kobayashi T. (2001). Real-time vibrational dynamics in chlorophyll a studied with a few-cycle pulse laser. Biophys. J..

[38] Yagasaki T., Saito S. (2008). Ultrafast intermolecular dynamics of liquid water: A theoretical study on two-dimensional infrared spectroscopy. J. Chem. Phys..

